# Comparison of wear on articular cartilage from polycarbonate-urethane and other implant biomaterials

**DOI:** 10.1177/09544119251412486

**Published:** 2026-01-23

**Authors:** Maha Ead, Andrea Dimitrov, Haoyang Li, Mohammadhamed Shahsavari, Kezhou Wu, Cameron Scott, Chester Jar, Jonelle Melissa Jn Baptiste, Nadr Jomha, Kajsa Duke, Lindsey Westover

**Affiliations:** 1 Department of Mechanical Engineering, University of Alberta, Edmonton, Canada; 2 Department of Surgery, University of Alberta, Edmonton, Canada; 3 Department of Medicine, University of Toronto, Toronto, Canada; 4 Department of Orthopedic Surgery, Shantou University Medical College, Shantou, China; 5 Department of Biomedical Engineering, University of Alberta, Edmonton, Canada; 6 Department of Civil & Environmental Engineering, University of Alberta, Edmonton, Canada

**Keywords:** orthopedics, talus prosthesis, articular cartilage, cartilage wear, biomaterials, polycarbonate-urethane

## Abstract

Talar replacement procedures offer good clinical outcomes for patients experiencing talar osteonecrosis with collapse. However, there is a potential for cartilage wear as the artificial talus prosthesis articulates against the native articular cartilage (AC) in the ankle joint. Therefore, this study investigated the wear of AC against candidate implant biomaterials with the aim of selecting an appropriate material for use in talar replacement procedures. Cobalt chrome alloy (Co-28Cr-6Mo), titanium alloy (Ti-6Al-4V), ultra-high molecular weight polyethylene (UHMWPE), industrial grade natural polyether ether ketone (PEEK), and polycarbonate-urethane (PCU) were tested against porcine AC submerged in bovine serum using an in vitro customized dual-motion wear testing setup. A total of 43,200 cycles at a frequency of 3 Hz were completed for each test. Both macroscopic and microscopic analyses were used to quantify cartilage wear using the Outerbridge and Osteoarthritis Research Society International (OARSI) clinical grading systems, respectively. In the macroscopic analysis, Ti-6Al-4V demonstrated the most AC wear, followed by Co-28Cr-6Mo, PEEK, UHMWPE, and PCU. In the microscopic analysis, PEEK demonstrated the most AC wear, followed by Ti-6Al-4V, Co-28Cr-6Mo, UHMWPE, and PCU. PCU demonstrated the least amount of AC wear compared to all other biomaterials and showed statistically insignificant differences with the control group (porcine cartilage-on-cartilage) in both macroscopic and microscopic inspections. These results suggest that PCU may be a suitable candidate material for coating talus implants as it demonstrated superior AC wear performance compared to the other biomaterials investigated in this study.

## Introduction

The talus is a critical bone in the human ankle joint and articulates with the tibia, fibula, calcaneus, and navicular bones. Talus bone injuries are therefore associated with high complication rates as well as poor outcomes due to limited treatment options.^1^ Additionally, its limited blood supply makes it prone to osteonecrosis which could potentially lead to talar collapse.^2^ Total ankle replacement (TAR) is a surgical treatment option for osteoarthritis where parts of the talus and tibia are replaced with prosthetic components.^3^ Although TAR has the potential to preserve ankle mobility, there are concerns with implant longevity and the need for revision surgeries.^3^ TAR is also not viable for patients with talar collapse as the talus bone cannot support the prosthesis.^4^ Another treatment procedure for osteonecrosis with talar collapse is tibiotalocalcaneal fusion, where the talus bone is fused to the tibia and calcaneus bones.^5^ Although this procedure is commonly performed for this disorder, it has significant limitations including restricted ankle joint motion and decreased weight-bearing capacity.^6^ Talar replacement is an alternative procedure where the damaged talus bone is substituted with an artificial prosthesis.^2^ This often involves using computed tomography (CT) scan data of the contralateral (uninjured) talus to design and create a customized implant,^7^ typically made of metal or ceramic.^2^ Alternatively, a non-customized implant based on a universal talus design may be used in cases where patients have injured both tali.^8^ Although talar replacement procedures yield good patient outcomes,^7^ there is a risk of cartilage wear as the implants articulate against adjacent ankle joint cartilage.^9^

Biomaterials used in implants must have good tribological properties for long-term effectiveness. Talar prostheses have been reported to be fabricated out of metals, including cobalt chrome alloy,^8,10–13^ stainless-steel (SS),^14,15^ and titanium alloys,^8,16^ as well as ceramic materials, such as alumina ceramic (Al_2_O_3_).^7,17,18^ Metals are commonly used implant materials as they demonstrate good biocompatibility.^19^ However, as the metals articulate against adjacent cartilage, there is a potential risk of releasing metallic ions and creating wear debris, resulting in inflammation.^20^ Additionally, metals have shown to significantly reduce articular cartilage (AC) thickness during in vitro wear tests.^21^ Other common biomaterials used in orthopedic implants are polymers, including ultra-high molecular weight polyethylene (UHMWPE) and polyether ether ketone (PEEK), due to their biocompatibility.^22^ The long-term effectiveness of UHMWPE implants is affected by the polymer’s limited wear resistance in total joint replacements. Cross-linking UHMWPE is done by manufacturers to improve wear resistance, however cross-linked UHMWPE has decreased mechanical strength, toughness, ductility, and fatigue resistance.^23^ Cross-linking methods can also lead to degenerative oxidation, however modern implants are stabilized with antioxidants to reduce this risk.^23^ PEEK is also used as an orthopedic biomaterial since it has good wear resistance, however it is limited by its high friction coefficient in cartilage articulations which limits its use in hemiarthroplasty applications.^24,25^

In hemiarthroplasties, due to articulation of the biomaterial on AC, a material with similar properties to AC is desired. Polycarbonate-urethane (PCU) is a synthetic material that has been employed in the orthopedic industry to better represent the physiological joint environment.^26^ It has a lower elastic modulus when compared to metals or ceramics and more closely resembles the biomechanical properties of native hyaline cartilage.^26^ A meniscal implant (NUsurface®, Active Implants, Tennessee) made of PCU has been implanted in patients after undergoing a meniscectomy and has shown to improve clinical outcomes in terms of alleviating pain and restoring normal joint biomechanics.^27^ PCU has also been used as a buffer between the acetabular shell and femoral head in total hip arthroplasties (TriboFit® Hip System, Joint Replacement Instrumentation Ltd., UK), enhancing patient clinical outcomes.^28^ In this study, we therefore hypothesize that coating the metal talus implants with PCU could potentially reduce the risk of AC wear after talar replacement surgery, thereby preventing joint degeneration.

Multiple wear testing configurations have been utilized to test the effects of biomaterial wear on AC in joint hemiarthroplasties, either at room or body temperature. The most common configuration is the pin-on-disk or a modified version of this method, including pin-on-ball and plane-on-cylinder.^29–36^ However, the pin-on-disk method does not accurately replicate the in vivo environment as constant forces and sliding speeds are applied, in contrast to physiological loads which vary throughout the gait cycle.^29–31^ Further, geometries and motion of the joint are often not represented well with the pin-on-disk configuration.^32–34^ For example, Trevino et al.,^29^ modified the pin-on-ball setup to represent the rolling and gliding motion at the tibiofemoral joint, however constant forces were used as opposed to the cyclic forces seen in vivo. To better replicate the joint in vivo, custom configurations have been created to represent hemiarthroplasties in specific joint environments.^35,36^ In the present study, a custom mechanical testing machine that couples rotational displacement and a sinusoidal compressive load to represent a more physiological loading scenario is used to evaluate AC wear when articulating against synthetic biomaterials.

Wear disrupts the structure and composition of cartilage leading to changes that can be visualized and quantified to measure the degree of wear.^9^ Wear can be effectively analyzed qualitatively with a histological analysis.^29,30,37–39^ Proteoglycan content can be visualized with Safranin-O/fast green stain and collagen content can be visualized with Picrosirius red or Masson Trichrome.^29,30,37–39^ The Osteoarthritis Research Society International (OARSI) grading system is used to grade the progression of osteoarthritis of articular cartilage at a microscopic level.^40^ The OARSI grading system is a simplified application of the OARSI histopathology grading/staging system that is a reliable and reproducible system.^41^ It demonstrates superior classification compared to other methods such as the Histologic Histochemical Grading System (HHGS) in terms of assessment of early disease.^40^ Further, the Outerbridge clinical grading system is a macroscopic evaluation for grading cartilage lesions and has been used by surgeons for communication purposes.^42,43^ It is considered the most widely used classification compared to other systems of describing chondral lesions by direct visualization.^43^ In the present study, grade scales are used to quantify the cartilage wear at both the microscopic and macroscopic levels using the OARSI and Outerbridge grading systems, respectively.

As there is a lack of quantitative wear measurements from experiments testing biomaterials against cartilage,^9,44^ this study aims to investigate the wear properties of AC against potential materials for the talus prosthesis using a customized configuration representing the ankle joint. A porcine model was chosen for this study since porcine cartilage closely matches the thickness of human cartilage and has been widely used in previous research.^45–48^ Bovine serum was employed as the lubricating medium, in accordance with previous wear investigations.^9,30,32,47,49^ All experiments were conducted at room temperature which is consistent with conditions reported in earlier studies.^30,47,50^

While previous experimental studies, such as those by Ajdari et al.,^9^ have compared the wear behavior of AC against cobalt chrome alloy, PCU, and alumina (Al_2_O_3_) ceramic, their use of a pin-on-disk setup with constant forces does not accurately capture the variable joint forces experienced in vivo. Additionally, Ajdari et al.^9^ used cartilage disks (10 mm in diameter) in their wear tests whereas the present study employs intact porcine condyle samples. Furthermore, numerical investigations by Liu et al.^51^ and Ruan et al.^52^ have examined the contact mechanics of various implant biomaterials using finite element models of the ankle joint, offering useful insight into their potential suitability. However, these simulations are static and therefore cannot characterize the AC wear that develops under cyclic, physiological loading conditions.

In a previous experiment, the wear effects of titanium alloy (Ti-6Al-4V), UHMWPE, and carbon fiber reinforced polyether ether ketone (CFR-PEEK) were tested against porcine AC submerged in phosphate buffered saline (PBS) using a customized dual-motion wear testing machine.^48^ The results demonstrated CFR-PEEK to have the most wear on AC compared to titanium alloy and UHMWPE, suggesting the use of titanium alloy and UHMWPE as implant materials over CFR-PEEK.^48^ In the present study, the same customized dual-motion wear testing setup is used to evaluate the wear behavior of porcine AC submerged in bovine serum when articulating against cobalt chrome alloy (Co-28Cr-6Mo), titanium alloy (Ti-6Al-4V), ultra-high molecular weight polyethylene (UHMWPE), industrial grade natural polyether ether ketone (PEEK), and polycarbonate-urethane (PCU) in order to select the most appropriate material for use in talar replacement surgeries.

## Methods

### Materials

A total of 26 tests were completed in this study: *n* = 3 of cartilage-on-cartilage, *n* = 5 of Co-28Cr-6Mo on cartilage, *n* = 5 of Ti-6Al-4V on cartilage, *n* = 4 of UHMWPE on cartilage, *n* = 4 of industrial grade natural PEEK on cartilage, and *n* = 5 of PCU on cartilage. The three cartilage-on-cartilage tests represented the control group. The UHMWPE and PEEK groups underwent one less test than the cobalt chrome alloy, titanium alloy, and PCU groups due to material availability.

The cobalt chrome, titanium, UHMWPE, and PEEK samples were cut into disks that were 40 mm in diameter and 10 mm in thickness. The surface roughness (R_a_) of the disk surfaces was processed to meet the ASTM F2033 standard for surgical implants of under 50 nm. All disk surfaces were polished to under 50 nm as depicted by a profilometer, excluding the UHMWPE disks which were polished to 1100–1600 nm (maximum achievable value). For the PCU samples, a layer of PCU (Bionate II® Grade 80A, DSM Biomedical, The Netherlands) was coated onto stainless-steel disks that were also 40 mm in diameter and a total of 10 mm in thickness (2–3 mm PCU layer and 7–8 mm stainless-steel layer). To achieve an R_a_ of at most 50 nm for these PCU-coated disks, a custom aluminum mold was manufactured and the surfaces were polished to under 50 nm as depicted by a profilometer. The stainless-steel disks were placed in the mold and an injection molder was used to inject a layer of PCU on top of the disks.

The articular cartilage samples used in this study were porcine medial femoral condyles and patellae that were harvested from the stifle joints of sexually mature pigs (5–6 months old). Porcine medial condyles have an average cartilage thickness of 1.12 mm^53^ which is within the range of average cartilage thickness in the human talus bone (0.94–1.62 mm)^54^ and were therefore deemed appropriate for this study. Ethical consent for the experimental use of animal tissue was obtained from the Research Ethics Office at the University of Alberta. The pigs were not specifically slaughtered for this study. Their hind limbs were obtained from a local deli (Delton Sausage House, Edmonton, AB, Canada) within 24 h of slaughtering. The medial femoral condyles and patellae were then extracted using a saw (Dremel®) and stored in Dulbecco’s Modified Eagle Medium F12 (Gibco^TM^) at 4°C until testing. All condyle samples were tested within 1 week of harvesting.

**Figure 1.**
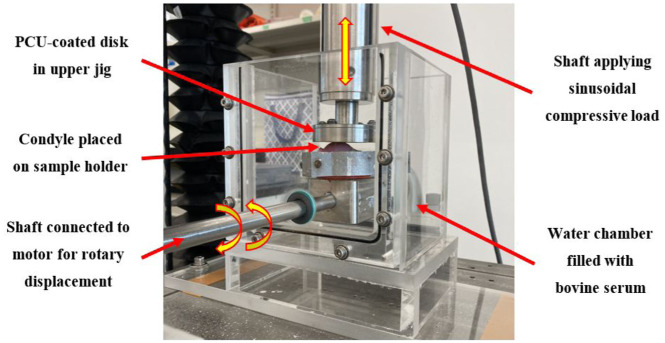
Custom wear testing setup.

The wear test was conducted with a customized wear testing setup utilizing a Bose Electroforce 3510 (Waters^TM^) and an electric motor. This setup provided both a rotational displacement (lower jig) and a sinusoidal compressive load (upper jig), as shown in Figure 1.^55^ The lower jig was 80 mm in diameter and the condyles were fixed to the plate using dental resin (Ortho-Jet^TM^) to prevent movement during testing. The disk samples were fit into the upper jig and screwed in place. For the control group cartilage-on-cartilage tests, the condyles were fixed to the lower jig and the patellae to the upper jig with dental resin.

Before testing, the water chamber was filled with approximately 1.5 L of diluted bovine serum. This solution acted as a lubricant to simulate synovial fluid in order to represent the physiological joint environment. The ISO 22622 standard was used to prepare this solution where 1 L of bovine calf serum (Hyclone^TM^) was diluted with 2.5 L of deionized water and 7 g of sodium azide were added to it. This created a 3.5 L solution of diluted bovine serum at a protein mass concentration of 20 g/L, which is consistent with several studies in the literature.^56–59^

### Mechanical wear testing

All tests were completed at room temperature (20°C–22°C). A cyclic compressive load of 30–160 N at 3 Hz was applied to the upper jig where the disk samples or patellae were held. Using the Hertzian contact model,^60^ the contact area was estimated to be 180 mm^2^ which, along with the compressive load, represented an average contact stress ranging from 0.19 MPa to 1 MPa.^48,60^ This contact model assumes that surfaces are frictionless, continuous, non-conforming, and elastic, and that the stresses and deformations outside the contact area are negligible.^61^ The compressive loads were chosen based on the reported range of peak joint stresses in vivo from 0.5 to 5 MPa.^61^ A rotation of ±10 degrees with a frequency of 3 Hz was applied to the lower jig where the condyles were held. A frequency of 3 Hz, as opposed to 1 Hz (commonly used in wear testing experiments), was used in order to simulate fast walking and for accelerated testing.^9,49,62^ A total of 43,200 cycles were completed for each test, which is similar to previous researchers analyzing cartilage wear from biomaterials in expedited test setups (under 50,000 cycles).^34–36,38^

### Cartilage wear assessment

To assess and quantify the degree of AC wear after the tests, both macroscopic and microscopic assessments were performed. For the macroscopic assessment, the Outerbridge clinical grading system was used to grade the samples.^42^ This grading system is based on a visual inspection of the condyles after the wear test and has a grading scale of 0–4. Lower grades (1–2) represent less damage to the cartilage with softening, swelling, and/or partial-thickness defects whereas higher grades (3–4) indicate more severe damage to the cartilage with fissures extending to the subchondral bone. A grade of 0 represents normal cartilage with no defects.^42^ To grade the samples after wear testing, pictures of the condyles were taken from five different views (anterior, posterior, medial, lateral, and top) using an iPhone XS/12Pro. The five pictures from each sample were grouped together as a set and these sets (each representing a different sample) were then randomly ordered and blindly graded by two researchers. In cases where the two researchers assigned different grades, the researchers discussed the findings to determine the final grade for that sample.

For the microscopic assessment, histology was performed and the OARSI grading system was used to grade the samples.^40^ The OARSI grading scale ranges from 0 to 6 where lower grades represent cartilage that is more intact and higher grades indicate greater cartilage damage including cartilage matrix loss and deformation. A grade of 0 represents normal cartilage with an intact surface and matrix morphology.^40^ To grade the samples, a 10 mm diameter osteochondral dowel was cored from the apex region of each condyle where maximum contact occurred. The bony base of the dowels was removed and each cartilage sample was cut into two halves. A section was then cut from each half and a microscopic slide was made so that each sample had two microscopic slides to be assessed. These slides were tested for proteoglycan content through Safranin O staining. Microscopic images of the slides were taken using a Zeiss Stemi 508 stereo microscope at 20× or 25× magnification. After all microscopic images were taken, they were randomly ordered and blindly graded by two researchers until a consensus was reached. It is important to note that only the descriptions of matrix appearance in the OARSI grading system^40^ were utilized and therefore the grade range was 0–4 in this assessment.

### Statistical analysis

Statistical analyses of quantitative data from both macroscopic and microscopic assessments were performed. Normal distributions were first assessed by checking if the skewness’s were less than 1 and if the standard deviations were less than half of the mean. Since both macroscopic and microscopic results showed data that was not normally distributed, the non-parametric Kruskal–Wallis tests were used. Post hoc pairwise comparisons were then conducted using Mann–Whitney tests with significance levels adjusted by the Benjamini–Hochberg correction. Statistical analyses were completed on IBM SPSS Statistics Version 27.0 (IBM Corporation, Armonk, NY, USA) and Microsoft Excel (Microsoft Corporation, Redmond, WA, USA).

## Results

**Figure 2.**
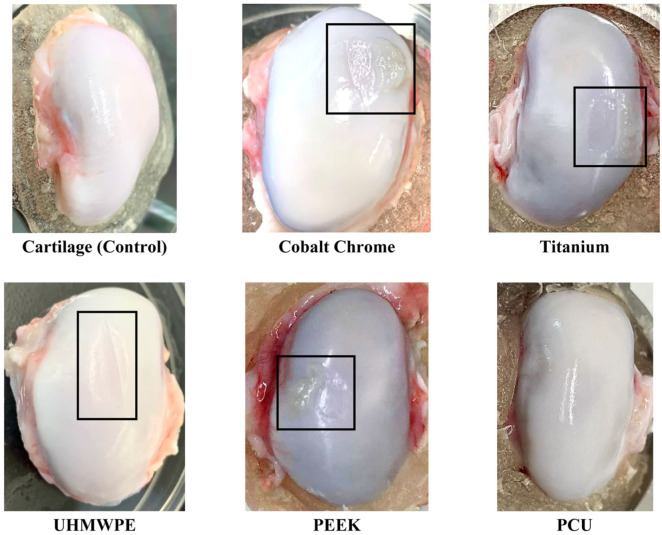
Example images of the condyle samples after wear testing which were used in the macroscopic assessment of cartilage using the Outerbridge clinical grading system. Damaged areas are indicated by a black box.

Figure 2 shows the top-view images of example condyles for each material after the wear test. These images were graded using the Outerbridge clinical grading system. All cartilage-on-cartilage tests received a grade of zero, indicating no wear and representing the control group. The average macroscopic wear grades (±standard deviation) of the material on cartilage tests were: cobalt chrome alloy (1.8 ± 0.45 with 95% CI: 1.24–2.36), titanium alloy (2.0 ± 0.00 with 95% CI: 2.00–2.00), UHMWPE (1.5 ± 0.58 with 95% CI: 0.58–2.42), PEEK (1.8 ± 0.50 with 95% CI: 0.95–2.55), and PCU (0.6 ± 0.55 with 95% CI: 0.08–1.28).

To compare the macroscopic wear grades between groups, a Kruskal–Wallis test was performed since the data was not normally distributed (skewness range = 0–2.236). The statistical power was estimated to be 0.893 using G*Power Version 3.1.9.7 (Heinrich-Heine-Universität Düsseldorf, Düsseldorf, Germany). The Kruskal–Wallis test indicated significant differences in macroscopic wear grades between groups (H(5) = 18.226, *p* = 0.003). Mann–Whitney tests (with Benjamini-Hochberg adjusted significance levels) were then completed for post hoc comparisons of mean ranks between all groups (Table 1). Results showed no significant differences in Outerbridge grades between any of the groups.

**Table 1.** Comparison between the mean ranks of the macroscopic Outerbridge grades.

Material comparison	*p*-value	Benjamini–Hochberg Alpha
Cartilage – cobalt chrome alloy	0.018	0.010
Cartilage – titanium alloy	0.018	0.007
Cartilage – UHMWPE	0.029	0.013
Cartilage – PEEK	0.057	0.023
Cartilage – PCU	0.196	0.033
Cobalt chrome alloy – Titanium Alloy	1.000	0.050
Cobalt chrome alloy – UHMWPE	0.524	0.040
Cobalt chrome alloy – PEEK	1.000	0.047
Cobalt chrome alloy – PCU	0.032	0.020
Titanium alloy – UHMWPE	0.167	0.030
Titanium alloy – PEEK	0.444	0.037
Titanium alloy – PCU	0.008	0.003
UHMWPE – PEEK	1.000	0.043
UHMWPE – PCU	0.159	0.027
PEEK – PCU	0.032	0.017

The sample size for each group is: *n* = 3 cartilage on cartilage, *n* = 5 cobalt chrome alloy on cartilage, *n* = 5 titanium alloy on cartilage, *n* = 4 UHMWPE on cartilage, *n* = 4 PEEK on cartilage, and *n* = 5 PCU on cartilage.

**Figure 3.**
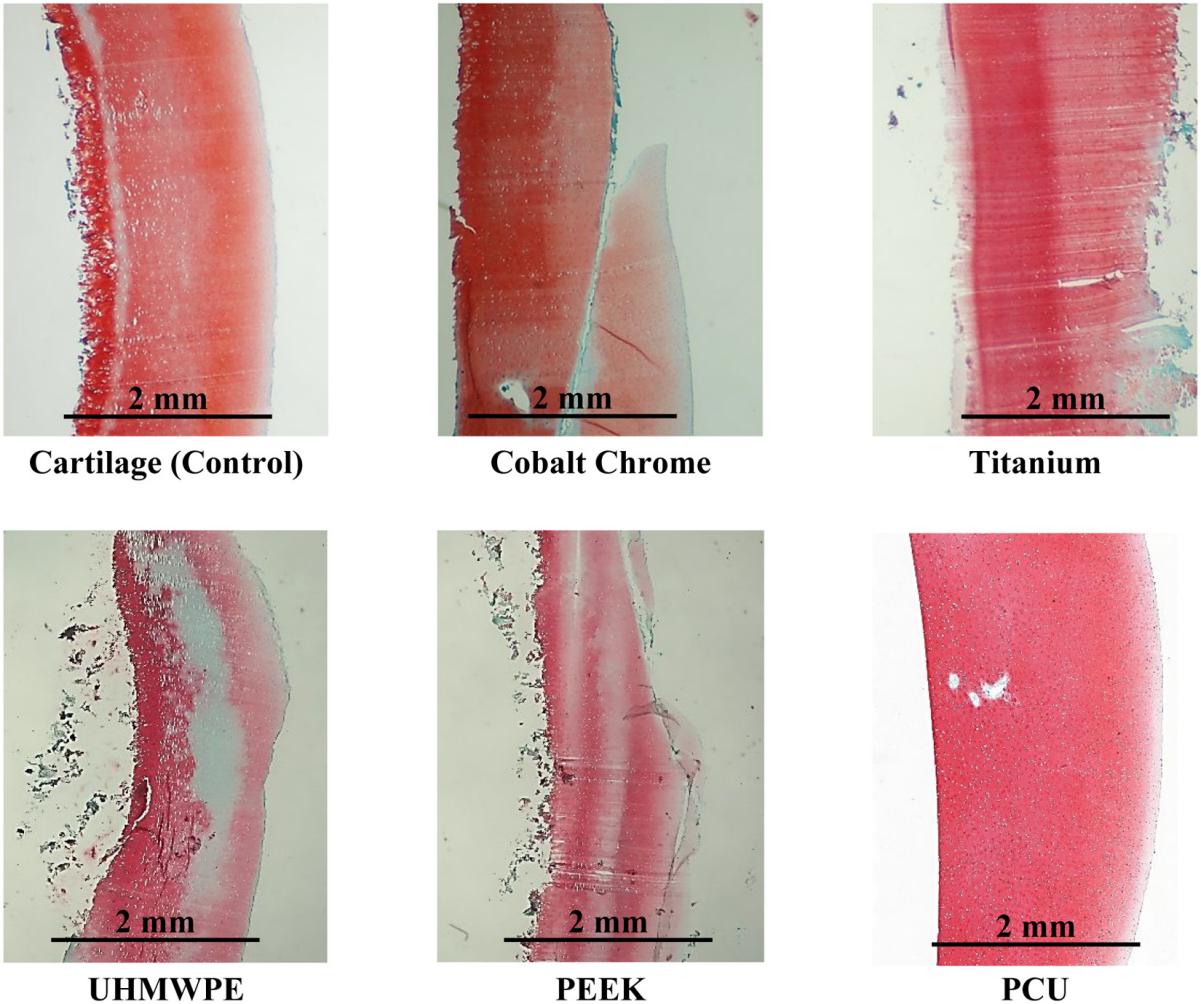
Example histological images of the condyle samples after wear testing which were used in the microscopic assessment of cartilage using the OARSI clinical grading system. The articulating surface is on the right side of each image. Scale bars are displayed on each sample.

For the microscopic assessment, example microscopic images for each material are displayed in Figure 3. Two slides were made for each sample; one slide from each half of the osteochondral dowels that were cored out of the condyles. The average OARSI grade (±standard deviation) for the cartilage-on-cartilage control group was 0.7 ± 0.52 (with 95% CI: 0.12–1.21) and the grades for the material on cartilage tests were: cobalt chrome alloy (3.0 ± 0.94 with 95% CI: 2.33–3.67), titanium alloy (3.2 ± 0.79 with 95% CI: 2.64–3.76), UHMWPE (1.9 ± 0.99 with 95% CI: 1.05–2.70), PEEK (3.3 ± 1.04 with 95% CI: 2.38–4.12), and PCU (0.5 ± 0.71 with 95% CI: 0.01–1.01).

The Kruskal–Wallis test was again performed to compare microscopic wear grades between groups since the data was not normally distributed (skewness range = 0.512–2.236). The statistical power was estimated to be 0.899 using G*Power software. The Kruskal–Wallis test showed significant differences in microscopic wear grades between groups (H(5) = 33.623, *p* < .001). Mann–Whitney tests (with Benjamini–Hochberg adjusted significance levels) were also performed to compare mean ranks of all groups and the results are summarized in Table 2. Results indicated that the OARSI grades of the cartilage-on-cartilage control group were significantly different from all the material groups, except for the PCU group. The PCU group OARSI grades were also significantly different from all the material groups.

**Table 2.** Comparison between the mean ranks of the microscopic OARSI grades.

Material comparison	*p*-value	Benjamini–Hochberg Alpha
Cartilage – cobalt chrome alloy	<0.001*	0.013
Cartilage – titanium alloy	<0.001*	0.010
Cartilage – UHMWPE	0.033*	0.033
Cartilage – PEEK	0.003*	0.023
Cartilage – PCU	0.547	0.043
Cobalt chrome alloy – titanium alloy	0.790	0.050
Cobalt chrome alloy – UHMWPE	0.048	0.037
Cobalt chrome alloy – PEEK	0.530	0.040
Cobalt chrome alloy – PCU	<0.001*	0.007
Titanium alloy – UHMWPE	0.011*	0.027
Titanium alloy – PEEK	0.737	0.047
Titanium alloy – PCU	<0.001*	0.003
UHMWPE – PEEK	0.025*	0.030
UHMWPE – PCU	0.003*	0.020
PEEK – PCU	<0.001*	0.017

Accounting for two slides per sample, the sample size for each group is: *n* = 6 cartilage on cartilage, *n* = 10 cobalt chrome alloy on cartilage, *n* = 10 titanium alloy on cartilage, *n* = 8 UHMWPE on cartilage, *n* = 8 PEEK on cartilage, and *n* = 10 PCU on cartilage.

* Statistically significant result.

## Discussion

In this study, a customized wear testing configuration was used to evaluate and compare the wear properties of porcine AC when articulating against several materials for potential use in a talus prosthesis. The materials included in this investigation were: cobalt chrome alloy, titanium alloy, UHMWPE, PEEK, and PCU. These materials were also compared with a cartilage-on-cartilage control group where minimal wear was expected since AC can endure high loads in vivo without damage.^63^ After all tests were completed, an assessment of the AC wear was conducted through macroscopic and microscopic analyses.

The macroscopic Outerbridge grades indicated that PCU caused the least amount of AC wear compared to all other biomaterials. The statistical analysis showed that PCU was not significantly different from the cartilage-on-cartilage control group or any of the material groups. In terms of the microscopic OARSI grades, PCU also demonstrated the least amount of AC wear and the statistical analysis showed that it was significantly different from all other material groups, except for the cartilage-on-cartilage control group. These results indicate that PCU may be an appropriate material for the fabrication of a talus prosthesis in comparison to the other materials reported in this study, since it presented with the lowest average Outerbridge and OARSI grades. UHMWPE had the second lowest average Outerbridge and OARSI grade.

These findings are consistent with the literature. In a study completed by Chan et al.,^44^ a pin-on-disk setup with reciprocating sliding motion immersed in PBS was used to quantify the wear on cartilage of four common hemiarthroplasty biomaterials alumina (Al_2_O_3_), cobalt chromium (CoCr) alloy, stainless steel (SS), and cross-linked UHMWPE. Wear was quantified using assays for total protein and superficial zone protein loss. UHMWPE resulted in the least amount of protein removed from the cartilage surface and the cobalt-chromium alloy resulted in the most amount of protein removed; although there were no significant differences between them.^44^ This aligns with the macroscopic results found in this study where UHMWPE had a lower average Outerbridge grade (1.5 ± 0.58) than the cobalt chrome alloy (1.8 ± 0.45), but the differences were not statistically significant. The low polymer stiffness of UHMWPE and the use of cross-linking may have contributed to the superior wear behavior of UHMWPE on cartilage, since cross-linking UHMWPE is hypothesized to reduce wear due to decreased plasticity and polymer orientation.^64^ In this study, the cobalt chrome alloy, titanium alloy, and PEEK disks were all polished to a surface roughness of under 50 nm, except for the UHMWPE disks which could only be polished to 1100–1600 nm. Despite this, the UHMWPE resulted in less cartilage wear than the cobalt chrome alloy, titanium alloy, and PEEK materials, suggesting that surface topography is only one of the factors that influence AC wear. The material properties of the opposing implant such as stiffness and viscoelasticity also contribute to AC wear.

PEEK implants were compared to cobalt chrome alloy implants in an in vivo animal study completed by Zhang et al.^65^ The macroscopic and histological results both indicated increased wear on AC from the cobalt chrome alloy in comparison with PEEK.^65^ In the current study, cobalt chrome alloy and PEEK demonstrated similar levels of wear from the macroscopic Outerbridge analysis: 1.8 ± 0.45 and 1.8 ± 0.50 respectively. From the microscopic analysis, the average OARSI grade was 3.0 ± 0.94 for the cobalt chrome alloy and 3.3 ± 1.04 for PEEK, indicating decreased wear on AC in the cobalt chrome alloy group. The current study used an in vitro configuration with a flat disk articulating against porcine condyles, whereas Zhang et al.^65^ used an in vivo study to investigate the effects of wear in a goat model. This difference in testing environments is a potential factor leading to the difference in wear results between cobalt chrome alloy and PEEK of our study compared to Zhang et al.^65^

In our study, PCU resulted in the least amount of AC wear compared to all other materials and showed no significant differences in macroscopic and microscopic grading when compared with the cartilage-on-cartilage control group. The average macroscopic Outerbridge grade for PCU was 0.6 ± 0.55 and zero for the cartilage-on-cartilage control group. As for the microscopic OARSI grades, PCU demonstrated an average of 0.5 ± 0.71, whereas the control group average was 0.7 ± 0.52. The superior wear performance of PCU can be attributed to its lower elastic modulus and viscoelastic properties compared to the other materials. Its biomechanical properties are similar to native cartilage, which could be a reason for the more favorable wear results.^26^ The lower stiffness of PCU allows it to deform more readily during articulation, increasing the contact area and distributing loads over a larger surface, which helps reduce peak contact pressures on the AC.^66^ Additionally, the reduced cartilage wear observed with PCU compared to the other polymers tested in this study (UHMWPE and PEEK) may be attributed to differences in surface hydrophilicity (wettability). PCU is inherently hydrophilic,^26,67,68^ whereas both UHMWPE and PEEK are hydrophobic materials.^67,69,70^ The hydrophilic nature of PCU promotes a more lubricated articulation with natural cartilage allowing PCU to mimic the physiological environment of a healthy synovial joint.^71^

The outcomes of the present study align with the findings of Ajdari et al.^9^ where they used a pin-on-disk setup to compare the wear on AC from cobalt chrome alloy, PCU, and alumina (Al_2_O_3_) ceramic. PCU performed the best and the cobalt chrome alloy demonstrated the most amount of AC wear.^9^ Liu et al.^51^ also found PCU to have superior contact characteristics in talus implants. Finite element simulations of ankle joint models in a neutral standing posture were conducted and the contact characteristics of the biological talus models were compared with cobalt chrome alloy talus models and PCU-coated talus models.^51^ Results indicated that coating talus implants with PCU reduces the contact pressure and increases the contact area in the surrounding AC.^51^ The PCU-coated models also had comparable contact characteristics to the biological models^51^ which further supports the findings of this study where the wear properties of the PCU group were not significantly different from the biological cartilage-on-cartilage control group. Furthermore, Ruan et al.^52^ conducted finite element simulations to investigate the use of PCU as a buffer layer for tibiotalar implant surfaces and showed that the peak stresses approached that of native cartilage. The results of these numerical studies along with the current study suggest that PCU may be a suitable candidate material for use in talus implants, since it demonstrated superior wear performance compared to the other materials investigated in this study.

This study has a number of limitations and considerations for future work. First, due to the intensity of the light microscope, cartilage cells were not visualized and the description of cell appearance on the OARSI grading scale was omitted. Analyzing the cell changes resulting from the wear experiment could have provided more insight on the wear rates of the materials. The coefficient of friction and volumetric wear rate are also important factors in analyzing the wear rate but were not quantified in the present study. Second, experimental setups can only act as screening tools for wear rates in vivo, since the physiological environment cannot be accurately duplicated. In vivo studies are needed to accurately represent the wear rates of these prosthetic materials on AC and would need to be completed in the future. Moreover, industrial grade (Röchling SUSTAPEEK) natural PEEK was used as there was difficulty in obtaining medical grade natural PEEK. In the future, medical grade materials should be used as they are approved for medical applications and would offer a more accurate representation of their performance. Also, a limited number of cycles (43,200 cycles) was used in this study and therefore does not simulate long-term in vivo conditions. More cycles should be incorporated in future testing for a more accurate representation of clinical life. Additionally, room temperature (20°C–22°C) was used to complete this experiment. Body temperature (37°C) should be used in the future to better replicate the physiological joint environment, since different temperatures may affect wear rates.^58^ Research has shown that the viscoelastic properties of polymers such as PCU are temperature-dependent, which may consequently influence the wear rate.^72^ Finally, this study provided a general comparison of the wear effects of several materials and, therefore a small sample size was used. However, in the future, sample sizes should be increased to improve reliability.

To summarize, this study investigated the wear of AC when articulating against several implant biomaterials with the aim of selecting the most suitable material for use in a talus prosthesis. A customized wear testing setup was used and porcine AC was tested for wear against cobalt chrome alloy, titanium alloy, UHMWPE, PEEK, and PCU. Both macroscopic and microscopic assessments indicated the least amount of AC wear when articulating against PCU. This suggests that PCU may be an appropriate material coating for talus prostheses.
